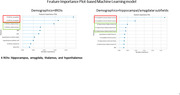# Volumes of specific substructures within the Amygdala and Hippocampus are impacted by brain amyloid‐β

**DOI:** 10.1002/alz.092024

**Published:** 2025-01-09

**Authors:** Leema Murali, Arnaud Charil, Anthonin Reilhac, Xin Qi

**Affiliations:** ^1^ Eisai Inc., Nutley, NJ USA

## Abstract

**Background:**

Reductions in medial temporal lobe (MTL) volume, particularly in the amygdala and hippocampus, are present in early Alzheimer's disease (AD). We explore the correlations between hippocampal and amygdalar subfield volumes and brain amyloid‐β (Aβ) accumulation using T1‐weighted structural MRI and amyloid PET data from ADNI and Eisai clinical trials.

**Method:**

We used FreeSurfer (v7.2.0) and a high‐resolution MRI‐based probabilistic atlas using Bayesian inference to measure volumes of hippocampal and amygdalar sub‐structures in one of Eisai’s clinical trial cohorts and ADNI (3113 and 1443 subjects), mostly with MCI due to AD (>80% and 45%, respectively). In Eisai’s clinical cohort, Aβ positivity was established through a concordance between PET visual assessment and Centiloids (CL), using a threshold of 32.21. In ADNI, Aβ positivity was defined by CL using a threshold of 20. We first evaluated correlations between baseline CL and volumes of hippocampus and amygdala substructures. For predicting brain amyloid burden, machine learning models, including stochastic gradient boosting, were applied in conjunction with cognitive and demographic information.

**Result:**

In Eisai’s clinical cohort, amygdalar subfields were more strongly correlated (p<0.05) with global amyloid levels than hippocampal subfields. Global amyloid is inversely associated with amygdala and hippocampal volumes, with the strongest correlations being with the amygdala accessory basal nucleus (left ‐0.41. right ‐0.40). This regional association with global amyloid is more significant than with the entire amygdala (left ‐0.36, right ‐0.35, p<0.05). Both the whole hippocampus (‐0.36) and its subfields (hippocampal GC‐ML‐DG (Granule Cell (GC) and Molecular Layer (ML) of the Dentate Gyrus (DG))‐body, subiculum‐body, and tail) showed equivalent correlations with global amyloid levels.

Machine learning models indicated that hippocampal/amygdalar subfields are better predictors of amyloid positivity than MTL volumes (increase in AUC from 66% to 70% (p<0.05)), which improved to 78% when adding ApoE4. Consistent with the results of the correlational analyses, the accessory‐basal nucleus, and hippocampal tail and fissure were among the top predictors in this model.

**Conclusion:**

This analysis emphasizes the importance of specific hippocampal and amygdalar substructures that are impacted by amyloid in early AD, with implications for staging and disease progression monitoring.